# An Unbiased Genome-Wide View of the Mutation Rate and Spectrum of the Endosymbiotic Bacterium *Teredinibacter turnerae*

**DOI:** 10.1093/gbe/evy027

**Published:** 2018-02-03

**Authors:** Marcus V X Senra, Way Sung, Matthew Ackerman, Samuel F Miller, Michael Lynch, Carlos Augusto G Soares

**Affiliations:** 1 Departamento de Zoologia, Universidade Federal de Juiz de Fora, Brazil; 2 Department of Bioinformatics and Genomics, University of North Carolina, Charlotte; 3 Biodesign Center for Mechanisms of Evolution, Arizona State University; 4 Departamento de Genética, Universidade Federal do Rio de Janeiro, Brazil

**Keywords:** mutation-accumulation (MA) assay, endosymbiosis, *Teredinibacter turnerae*, drift-barrier hypothesis

## Abstract

Mutations contribute to genetic variation in all living systems. Thus, precise estimates of mutation rates and spectra across a diversity of organisms are required for a full comprehension of evolution. Here, a mutation-accumulation (MA) assay was carried out on the endosymbiotic bacterium *Teredinibacter turnerae*. After ∼3,025 generations, base-pair substitutions (BPSs) and insertion–deletion (indel) events were characterized by whole-genome sequencing analysis of 47 independent MA lines, yielding a BPS rate of 1.14 × 10^−9^ per site per generation and indel rate of 1.55 × 10^−10^ events per site per generation, which are among the highest within free-living and facultative intracellular bacteria. As in other endosymbionts, a significant bias of BPSs toward A/T and an excess of deletion mutations over insertion mutations are observed for these MA lines. However, even with a deletion bias, the genome remains relatively large (∼5.2 Mb) for an endosymbiotic bacterium. The estimate of the effective population size (*N*_e_) in *T. turnerae* is quite high and comparable to free-living bacteria (∼4.5 × 10^7^), suggesting that the heavy bottlenecking associated with many endosymbiotic relationships is not prevalent during the life of this endosymbiont. The efficiency of selection scales with increasing *N*_e_ and such strong selection may have been operating against the deletion bias, preventing genome erosion. The observed mutation rate in this endosymbiont is of the same order of magnitude of those with similar *N*_e_, consistent with the idea that population size is a primary determinant of mutation-rate evolution within endosymbionts, and that not all endosymbionts have low *N*_e_.

## Introduction

Spontaneous mutations contribute largely to the input of genetic variation into living systems, and compose a major force in driving the evolutionary process. Accurate estimates of the spontaneous rate and spectrum of mutations across a large number of species are required to create a better comprehension of evolutionary patterns (Lynch et al. 2016). The least biased approach for mutation-rate estimation is the mutation-accumulation (MA) assay (Denver 2009; Lee et al. 2012; Sung et al. 2012). Initially proposed by Mukai (1964), an MA experiment uses continuous individual passages of several lineages derived from a single ancestral colony. The reduction in effective population size, *N*_e_, reduces the efficiency of selection on spontaneous mutations, allowing for the accumulation of all but the most deleterious mutations. Genome-wide sequencing of multiple MA lines has generated unbiased estimates of the rate and spectrum of spontaneous mutations across eukaryotes and bacteria (Lynch et al. 2016). Mutation rates vary ∼1,000-fold across species (∼10^−7^–10^−10^ mutations per site per generation) and the evolution of mutation rate has been linked to the population size of the organisms (Lynch 2010a; Sung et al. 2012, 2016). Specifically, the effective population size determines the power of random genetic drift, which defines the lower limit to which selection can promote reduction of deleterious mutation rates (Lynch 2008, 2010b; Sung et al. 2012; Lynch et al. 2016).

Although the number of MA genome-wide sequencing experiments has increased dramatically in the last few years, these experiments have been restricted to free-living model organisms. Endosymbiotic bacteria that are phylogenetically diverse (Moya et al. 2008), ecologically ubiquitous, and central to host evolution (Margulis and Bermudes 1985; Schink 1997; Minic and Hervé 2004; Zientz et al. 2004; Croft et al. 2005; Stewart et al. 2005; Kneip et al. 2007) have not been subject to such assays, mostly because of methodological reasons. As consequence of their long-term and intimate intracellular associations, endosymbionts are thought to display elevated mutation rates in comparison with their free-living close relatives (Moran 1996; Itoh et al. 2002; Woolfit and Bromham 2003) and eroded genomes (often <1 Mb coding for ∼300 genes), with the loss of essential cell functions forcing most of them to rely on host cells for survival (McCutcheon and Moran 2011). This pattern of evolution is thought to start just after the establishment of the host-restricted association (Moran 1996). Such a lifestyle can force the bacterium through constant population bottlenecks (between-generation host transmissions) and drive a reduction of the *N*_e_ of the symbiont (now limited to the host abundance). Under this view, the increased power of genetic drift (which is inversely related to *N*_e_) reduces the efficiency of purifying selection at removing slightly deleterious mutations, leading to a deleterious pattern of genome evolution (Lynch et al. 2016) through processes associated with Muller’s ratchet (Moran 1996).

Here, we have performed an MA experiment on the endosymbiotic bacterium *Teredinibacter turnerae*. This cellulolytic and nitrogen-fixing γ-proteobacterium colonizes specialized structures called glands of Deshayes in both demibranchs of mollusks of the family Teredinidae (Bivalve: Pholadoidea). *Teredinibacter turnerae* supplies cellulolytic enzymes and nitrogen compounds (Carpenter and Culliney 1975; Trytek and Allen 1980; Gallager et al. 1981; Waterbury et al. 1983; Distel et al. 1991, 2002; Lechene et al. 2007) that allow their hosts to feed on wooden material during their juvenile and adulthood stages. The complete genome sequencing of this endosymbiont has revealed that ∼7% of its genome is devoted to proteins involved in the biosynthesis of secondary metabolites (Yang et al. 2009), including antibiotics, suggesting that this bacterium is highly involved in the synthesis of bioactive metabolites required for the host survival (Trindade-Silva et al. 2009; Han et al. 2013; Elshahawi et al. 2013). Yet, despite its intracellular life-style, this bacterium can be cultivated independent of its host (Waterbury et al. 1983), allowing an evaluation of the mutation pattern of this unique intracellular bacterium by MA assays.

## Materials and Methods

### MA Assay

The *T. turnerae* strain used in this work (CS30) was isolated from *Neoteredo reynei* (Teredinidae) sampled in a Mangrove in Rio de Janeiro, Brazil (Trindade-Silva et al. 2009). Starting from a single cell, ∼100 independent MA lines were derived. Every 2 days, a single individual colony from each MA line was isolated and transferred to a fresh solid BMS (Trindade-Silva et al. 2009) and incubated at 30 °C over the course of the experiment. The imposed bottlenecking process ensures that mutations accumulate in an effectively neutral fashion, as demonstrated by Kibota and Lynch (1996). Generation time was estimated monthly using an entire colony from 12 randomly selected MA lines, transferred to 1× PBS saline buffer, vortexed, serially diluted, and replated for CFU counting. The generation time for each MA line was calculated using the harmonic mean of the cell divisions per transfer over the course of the experiment. After an average of 3,025 generations, the MA assay was concluded and the genomic DNA of the MA lines were extracted using the wizard DNA extraction kit (Promega) to Illumina library standards.

### Sequencing and Alignment

101-bp paired-end Illumina (Illumina Hi-Seq platform) sequencing was applied to randomly selected 47 *T. turnerae* MA lines. The coverage depth of each MA line was ∼100× and the average library fragment size (distance between paired end reads) was ∼175 bp. The paired-end reads from each MA line were individually mapped against the reference genome *T. turnerae* T7901 (Yang et al. 2009; available at https://www.ncbi.nlm.nih.gov) using two different mappers: BWA v0.7.4 (Li and Durbin 2009) and NOVOALIGN v2.08.02 (available at www.novocraft.com). The generated pileup files were converted to SAM format using SAMTOOLS v0.1.18 (Li 2011). We used in-house perl scripts to parse the alignments and to produce forward and reverse mapping information at each site, resulting in a configuration of 8 numbers for each line (A, a, C, c, G, g, T, t) corresponding to the number of reads mapped at each genomic position in the reference sequence. A separate file was also generated to display sites that had insertion–deletion (indels) mutation calls from the two alignment algorithms.

### Mutation Calling

Base-pair substitutions (BPSs) identification was carried out as previously described (Sung et al. 2015) using a consensus approach to identify putative mutations by comparing each individual MA line (focal line) against the consensus of all the other MA lines. Indels were identified as in Lynch (2010a) and briefly, followed the criteria: 1) At each position, a consensus indel requires 30% of the reads spanning a position in a line to indicate the same indel (size and motif); 2) Each consensus indel requires a minimum of two forward and two reverse reads spanning the indel; 3) At a single site, any identical indel event that is shared across >50% of the lines is considered either a progenitor indel that existed prior to the initiation of the MA line, or a genome assembly error, and not included in the final indel list; 4) BWA and NOVOALIGN alignment algorithms must both independently identify the site as a putative indel. The use of two separate alignment algorithms ensures that algorithm-specific alignment errors are not responsible for false-positive mutation calls. Short-read mapping algorithms have difficulties mapping indel events >10 bp, so we fed the BWA and NOVOALIGN alignment output into PINDEL (Ye et al. 2009), a short-read realignment algorithm used to identify indels through paired-end information. Our criteria for PINDEL for indels included the following: 1) Each indel requires a minimum of six forward and six reverse reads indicating the indel, with a minimum of 20 reads overall supporting the indel call; and 2) any indel shared across >50% of the lines is considered a progenitor indel that existed prior to the initiation of the MA line, and excluded from the final indel list.

### Mutation Rate Calculation

The base-substitution (BPS) mutation rate per site per generation (μ_bs_) for each MA line is estimated as equation (1):

(1) $$\mu_{bs}=\frac{m}{nT},$$

where *m* is the number of observed base substitutions, *n* is the number of nucleotide sites analyzed, and *T* is the total number of generations in the MA assay. The standard error for each MA line was calculated using equation (2):

(2) $${SE}_{x̄}=\sqrt{\frac{\mu_{bs}}{nT}}.$$

The total standard error of the BPS mutation rate is estimated by equation (3):

(3) $${SE}_{pooled}=\frac{s}{\sqrt{N}},$$

where *s* represents the standard deviation of the mutation rates across all lines, and *N* is the number of lines analyzed.

The same calculation was used to calculate indel mutation rate, with *μ*_bs_ replaced with μ_indel_.

### Effective Population Size

Under the assumption of neutrality, *T. turnerae’s N*_e_ was indirectly derived from the average nucleotide heterozygosity at silent-sites from complete and draft genomes of *T. turnerae* strains publicly available in the GenBank (http://www.ncbi.nlm.nih.gov/genbank/) as described (Sung et al. 2012).

### Multinucleotide Mutations Probability Estimation

If we assume mutations are random with respect to their position, the chance of occurrence of one or more adjacent mutations in any giving genome can be estimated. The expected probability of occurrence of multinucleotide mutations (MNMs) within window sizes of 20, 50, or 100 nt in the genome of *T. turnerae* MA lines, is the number of observed BPSs (779) divided by the genome size (5.19 × 10^6^), and by the number of MA lines (47), and multiplied the corresponding window sizes (20, 50, or 100 nt). The chance of occurrence of one MNM in at least one window in a single MA line is then (Schrider et al. 2011):

(4) $$1-\text{cdf}{\left(1,\;\lambda \right)}^{n},$$

where cdf is the cumulative distribution function for a Poisson process, λ is the per-window rate, and *n* is the number of windows in the genome. The probability to observe even one MNM in the genome of these MA lines was <0.01 for all tested window lengths (only data for 50 nt shown, supplementary table S4, Supplementary Material online).

## Results

### MA Assay

The *T. turnerae* strain CS30 used in this work was originally isolated from a *N. reynei* (Teredinidae, Bivalve) host sampled in a mangrove in Rio de Janeiro, Brazil (Trindade-Silva et al. 2009). 47 MA lines of *T. turnerae* underwent an average of 3,025 generations of parallel single-individual passages from the ancestral CS30 strain on solid basal medium with sucrose (BMS) at 30 °C (Trindade-Silva et al. 2009). By the end of this process, a number of MA lines were displaying smaller colony sizes, consistent with reduced fitness from the accumulation of deleterious load (Kibota and Lynch 1996). The *T. turnerae* MA lines were subjected to 101-bp paired-end high-throughput whole-genomic sequencing (Illumina Hi-Seq platform); the resulting reads were mapped against the *T. turnerae* T7901 (NC_012997) reference genome; and mutations were identified as previously described (Sung et al. 2015). Spontaneous-mutation data are summarized in table 1 (with further details in supplementary tables S1–S3, Supplementary Material online). As in other MA experiments with other organisms (Denver et al. 2012; Lee et al. 2012; Sung et al. 2012), the number of BPSs in coding regions (*χ*^2^ = 5.748, df = 1, *P* = 0.016) and the expected ratio of nonsynonymous to synonymous mutations (*χ*^2^ = 3.220, df = 1, *P* = 0.070) are not significantly different from random expectations. Because a large proportion of nonsynonymous mutations are expected to be removed by natural selection if effective, a random distribution of nonsynonymous and synonymous mutations indicates that selection played a minimal role on the mutation process during this MA experiment.

**Table 1:** Summarized Mutation-Accumulation (MA) Assay Data

	MA Lines (*n*)	Gen.	BPSs	Indels	Total Mutation Events	BPSs Rate (×10^−10^)/Site/Generation	BPSs Rate (×10^−3^)/Genome Replication	Ts/Tv
Total	47	3,025	779	106	885	—	—	—
Ave.	—	—	16.57	2.26	18.83	11.4	5.59	3.48
Max	—	—	28	6	34	19.8	9.26	17
SEM	—	—	0.81	0.24	1.05	0.55	1.61	0.47

NOTE.—Ave., average; Max, maximum; SEM, standard error of the mean; Gen., number of generations; indels, insertion/deletion events; Ts/Tv, transition/transversion ratio.

### Mutation Rate and Distribution of Mutations

**Fig. 1.**
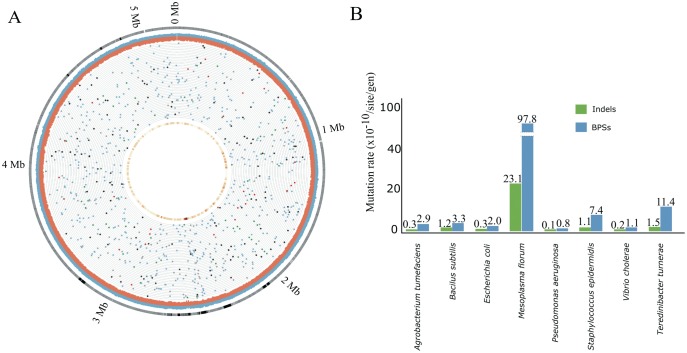
—(*A*) Distribution of mutations, BPSs, and indels mapped in the 47 *Teredinibacter turnerae* CS30 MA lines. From the outer ring to inner ring scaled to total genome size: gene density (grey), significantly elevated (1 kb blocks >2 standard deviations from the mean) G/C (blue) or A/T content (red), position of base substitutions in each line (black—intergenic substitution; grey—synonymous substitution; blue—nonsynonymous substitution; red—insertions; green—deletions), and base-substitution density (25 kb blocks, red > orange > yellow). Circos plot (Krzywinski et al. 2009) was used to create this figure. Please access the online version for color information on this figure. (*B*) Mutation rates (BPSs and indels per site per generation) across different bacterial species. The data for this analysis were extracted from Sung et al. (2016).

A total of 885 spontaneous mutations accumulated within the genomes of the 47 tested *T. turnerae* MA lines, of which 779 (88.1%) are BPSs and 106 (11.9%) are indels (fig. 1*A*, table 1, supplementary tables S1–S3, Supplementary Material online). The genome-wide BPSs rate of 1.14 × 10^−9^ (standard error of the mean [SEM] = 0.55) per site per generation, or 5.59 × 10^−3^ (SEM = 1.61) mutations per genome per generation, and the indel rate 1.55 × 10^−10^ (SEM = 0.16) events per site per generation are among the highest within free living and facultative intracellular bacteria (fig. 1*B*). Although the BPSs are distributed randomly with respect to function, the distribution of mutations with respect to chromosomal position is not homogeneous. Similar to *Escherichia coli* (Foster et al. 2013), a peak of mutations is observed near the replication terminus located at chromosome position ∼2.5 Mb (Yang et al. 2009). As shown in supplementary figure S1, Supplementary Material online, a 50-kb bin containing the replication terminus of *T. turnerae* accumulated significantly more BPSs than the other bins elsewhere in the genome of this bacterium (one sample-*t* test = 36.50, df = 103, *P* = 3.15 × 10^−37^; supplementary fig. S1, Supplementary Material online). Moreover, we noticed an excess of adjacent mutations (within single MA lines) corresponding to 21 BPSs and 16 indels (4.2% of the total de novo mutations) arising within <50 nucleotides from each other (supplementary table S4, Supplementary Material online). Under the assumption that mutations are randomly distributed across the genome, the probability of the occurrence of MNMs (Schrider et al. 2011), defined here as two or more mutations within windows of 50 nucleotides is expected to be extremely low (*P* = 1.96 × 10^−4^). Yet, MNMs are observed in over a third (34.0%) of the MA lines. Most clustered mutations are found as pairs, with a single instance of three closely neighboring mutations in MA line 29 (supplementary table S4, Supplementary Material online). Our data do not allow us to determine whether these MNMs arose as a single event, but, if not, they had at least occurred in a short period of time (during the course of this MA assay). These data are consistent with the idea that spontaneous mutations may not be independent with respect to position (Amos 2010; Schrider et al. 2011; Sung et al. 2015).

### Mutation Spectrum

**Fig. 2.**
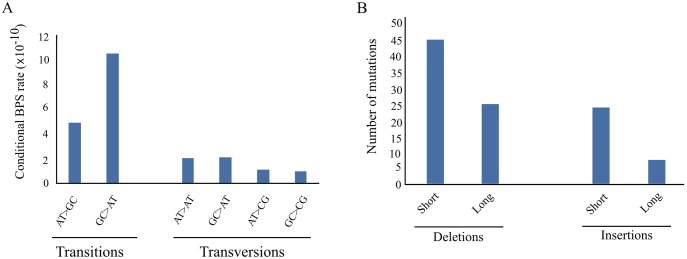
—Mutation spectrum of *Teredinibacter turnerae* MA lines. (*A*) Conditional BPSs rate normalized to the number of AT or GC base pairs in the genome. (*B*) Indels. Short indels are defined here as indels ranging from 1 to 3 bp, whereas long indels are ≥ 4 bp.

Of the total 779 BPSs, 71.6% (558/779) are transitions (supplementary table S1, Supplementary Material online), yielding a Transition/Transversion (Ts/Tv) BPS ratio of 3.48 (SEM 0.47; table 1). The observed transition bias is consistent with that observed in other MA experiments (Ochman 2003; Lynch 2010b). BPSs toward A/T (fig. 2*A*) account for 68.4% (533/779) of all BPSs, which is also commonly observed in other bacterial systems (Lynch et al. 2008; Denver 2009; Keightley et al. 2009; Hershberg and Petrov 2010; Hildebrand et al. 2010; Sung et al. 2012), and thought to be linked to the high observed rate of spontaneous deamination of cytosine and the conversion of guanine to 8-oxo-guanine (Duncan and Miller 1980). Considering deletion mutation events, 64% (47/73) are short (1–3 bp) and 36% (26/73) long (4 bp or more; fig. 2*B*), including a 12,581-bp long deletion in MA line 56. Short insertions account for 75% (25/33) of all insertions (fig. 2*B*). Overall, there is a significant excess of deletions (68.8%, 73/106) over insertions in this MA experiment (*χ*^2^ = 15.09, df = 1, *P* = 0.10 × 10^−3^), with deletion accounting for a loss of 9.89 × 10^−2^ nucleotides per generation per MA line (175.93-fold higher than the gain by insertions). These data are consistent with a general deletion bias observed in bacteria (Mira et al. 2001; Kuo and Ochman 2009; Koskiniemi et al. 2012).

### Effective Population Size (*N*_e_) Estimates

**Fig. 3.**
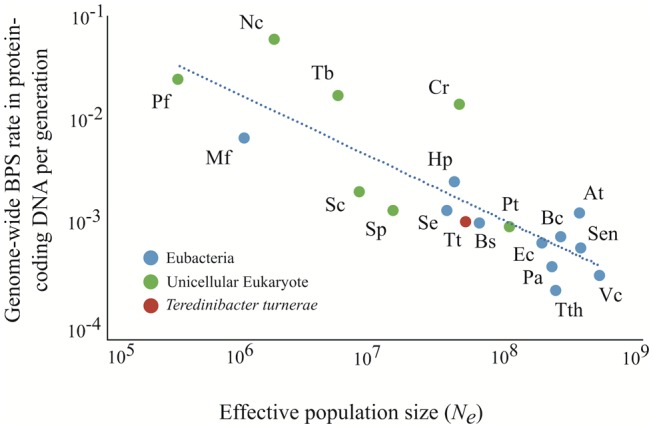
—Average total genome-wide BPSs rate in protein coding DNA per generation as a function of *N*_e_. Trend line *F*(*x*) = 64.35×^−0.59^ (*r*^2^ = 0.693). The data for this analysis were extracted from Lynch et al. (2016). Labels: Eubacteria: At, *Agrobacterium tumefaciens*; Bs, *Bacillus subtilis*; Bc, *Burkholderia cenocepacia*; Ec, *Escherichia coli*; Hp, *Helicobacter pylori*; Mf, *Mesoplasma florum*; Pa, *Pseudomonas aeruginosa*; Sen, *Salmonella enterica*; Se, *Staphylococcus epidermidis*; Tth, *Thermus thermophila*; Tt, *Teredinibacter turnerae*; and Vc, *Vibrio cholerae*. Unicellular Eukaryotes: Cr, *Chlamydomonas reinhardtii*; Nc, *Neurospora crassa*; Pf, *Plasmodium falciparum*; Pt, *Paramecium tetraurelia*; Sc, *Saccharomyces cerevisiae*; Sp, *Schizosaccharomyces pombe*; Tb, *Trypanosoma brucei*.

The effective population size (*N*_e_) is inversely proportional to the power of random genetic drift, which has been suggested to have an influence on the evolution of mutation rate (Lynch 2010a; Sung et al. 2012; Lynch et al. 2016). According to the drift-barrier hypothesis (DBH), the genome-wide mutation rate affecting fitness (estimated by the total protein coding nucleotides) is expected to be inversely related to *N*_e_. In haploid organisms and under the assumption of neutrality, *N*_e_ can be estimated using the formula *π_S_* = 2*N*_e_μ_bs_, where *π_S_* is equal to the variation at silent sites among natural isolates, and μ_bs_ is the BPSs rate per site per generation. *Teredinibacter turnerae*’s *π_S_* was measured by comparing silent-site diversity in draft *T. turnerae* genomes (deposited in GenBank) according to Sung et al. (2012). By substituting the estimated μ_bs_ (1.14 × 10^−9^ per site per generation) in the formula, we estimate *N*_e_ to be ∼4.5 × 10^7^. As shown in figure 3, this *N*_e_ is slightly lower than comparable measures in most free-living bacteria (e.g., *E. coli N*_e_ is estimated to be on the order of 10^8^ [Sung et al. 2016]). Moreover, the mutation rate in this endosymbiont is quite similar to rates in other species with similar *N*_e_ (fig. 3), reinforcing the idea that *N*_e_ (Sung et al. 2012) has a large effect in determining the mutation rate evolution in endosymbionts as in other species (McCutcheon and Moran 2011).

## Discussion

Endosymbiotic bacteria have a unique lifestyle of coexistence with their hosts, and the forces driving their evolution differ from those of free-living bacteria and have a profound impact on their patterns of genome evolution (Wernegreen 2015). From evolutionary theory, one may predict that endosymbionts are subject to relaxed purifying selection on metabolic pathways that are redundant in host’s genomes (Moran 2003), resulting in a reductive genome evolution, and increased fixation of slightly deleterious mutations by genetic drift due to *N*_e_ reduction caused by population bottlenecks intrinsic to the host-restricted lifestyle (for more exhaustive review on this topic, see Wernegreen 2015).

Confirming theoretical predictions on the role of the endosymbiotic lifestyle in genome evolution, prior mutation studies using phylogenetic comparisons and recent genomic data from a number of insect endosymbiotic species have brought to light that these organisms have rapid sequence evolution in comparison to closely related free-living bacteria (Moran 1996; Itoh et al. 2002). In addition, they often display severely eroded genomes (Kuo and Ochman 2009), where all but essential genes for the maintenance of the host association are lost. An extreme example of this process is “*Candidatus* Tremblaya princeps” that has a complete genome length of ∼139 kb coding only for 110 genes (López-Madrigal et al. 2011; McCutcheon and Moran 2011).

In MA lines of the endosymbiont *T. turnerae*, we found a rate of BPSs (1.14 × 10^−9^ per-site per generation) higher than those observed in most free-living and facultative intracellular pathogenic bacteria and of the same order of magnitude of other nonfree-living bacteria such as *Buchnera aphidicola* (∼4 × 10^−9^ per site per generation; Moran et al. 2009). The observed indel rate in the present study (1.55 × 10^−10^ events per site per generation) is also one of the highest among bacteria (with established indel rates based on whole-genome sequencing data generated after mutation accumulation assay; fig. 1*B*). Furthermore, we observed a strong deletion bias (*χ*^2^ = 15.09, df = 1, *P* = 0.10 × 10^−3^), with 2.2× more spontaneous deletion events than insertion events, resulting in a loss of 9.83 × 10^−2^ nucleotides per generation per MA line. This observation suggests that genome erosion might be strong and have a role in *T. turnerae* evolution.

Although *T. turnerae* exhibits mutational properties that are similar to other endosymbionts (higher mutation rate and strong deletion bias), *T. turnerae* retains a relatively large genome (∼5.2 Mb), comparable in size to many free-living bacteria such as *E. coli* (Blattner 1997), *Pseudomonas aeruginosa* (Stover et al. 2000), and *Bacillus subtilis* (Kunst et al. 1997). Because genomic erosion is a time dependent process, it may be suggested that this symbiotic relationship has arisen recently. Yet, fossil records reveal that this is not a recent association, with the family Teredinidae dating back to lower Cretaceous (100–145 million years [Myr]; Lopes et al. 2000), similar to the 150 Myr proposed for the symbiotic association between aphids and *B. aphidicola* that has a genome size of 0.6 Mb (Bennett and Moran 2014). However, population data show that the measured effective population size of *T. turnerae* (∼4.5 × 10^7^) lies between the *N*_e_ measured in the free-living bacteria *B. subtilis* (10^7^) and *E. coli* (10^8^) (Sung et al. 2012), suggesting that *T. turnerae* may not be subject to heavy bottlenecking events such as those theoretically predicted to occur along the life history of host-restricted endosymbionts (Wernegreen 2015). Thus, although *T. turnerae* exhibits a statistically significant deletion bias, it has not experienced the large-scale genome erosion usually found in endosymbionts, presumably because of more effective selection in removing deletions from population. Our findings are consistent with the expectation that effective population size has a large influence on the mutation rates (both indel and BPSs; Sung et al. 2012, 2016) and genome size (Moran 2003; Kuo and Ochman 2009; McCutcheon and Moran 2011; Wernegreen 2015).

*Teredinibacter turneae* is an intracellular bacterium that can be cultivated under in vitro conditions. Moreover, based on morphological data and the high sequence identity of the 16S rDNA from *T. turnerae* isolated from many Teredinidae species from unrelated geographical regions, this bacterium might be subject to horizontal transfer across hosts (Waterbury et al. 1983; Distel et al. 1991). In fact, many endosymbiotic bacteria can be horizontally transmitted between hosts (Sandström et al. 2001; Haselkorn et al. 2009; Russell et al. 2009; Duron et al. 2010), and some intracellular opportunistic pathogenic bacteria such as *P. aeruginosa* (LaBauve and Wargo 2012) are capable of surviving in the environment as free-living organisms. Through similar mechanisms, these endosymbionts might be able to maintain large *N*_e_ during their life cycles. Thus, we propose that the endosymbiont *T. turnerae* is maintained in large enough populations when transferred from one generation to another, with selection acting efficiently enough to counteract a deletion bias and to maintain mutation rates at levels that lie between those of free-living bacteria and endosymbiotic bacteria that undergo frequent bottlenecking.

## Supplementary Material

Supplementary data are available at *Genome Biology and Evolution* online.

## Supplementary Material

Supplementary Data
