# Supplementary figures and images for: An Unbiased Genome-Wide View of the Mutation Rate and Spectrum of the Endosymbiotic Bacterium Teredinibacter turnerae
Source: Genome Biol Evol. 2018 Feb 3;10(3):723–30. doi: 10.1093/gbe/evy027 (PMC5833318; doi:10.1093/gbe/evy027)

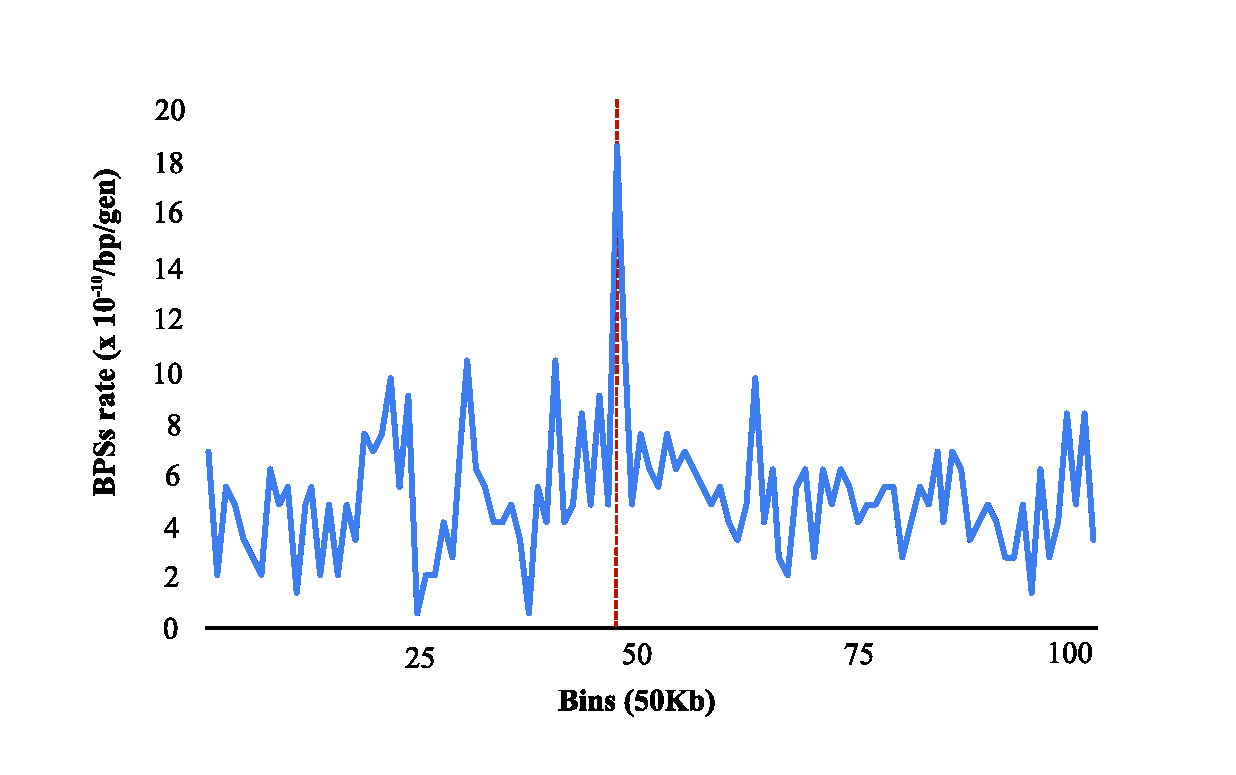

Supplement: Supplementary Data [file evy027_supp.zip › FigS1-submit.tif]
